# Chimeric antigen receptor (CAR) -T cell therapy treatment Richter's transformation

**DOI:** 10.3389/fonc.2025.1591360

**Published:** 2025-06-09

**Authors:** Shaomei Feng, Peihao Zheng, Haidi Liu, Meiling Sun, Yuelu Guo, Lixia Ma, Rui Liu, Zhonghua Fu, Fan Yang, Xiaoyan Ke, Kai Hu

**Affiliations:** ^1^ Department of Myeloma and Lymphoma, Beijing GoBroad Boren Hospital, Beijing, China; ^2^ Department of Lymphoma and Myeloma Research Center, Beijing GoBroad Hospital, Beijing, China

**Keywords:** CAR-T cell therapy, leukemia, disease progression, survival analysis, Richter’s transformation, B-NHL

## Abstract

**Background/purpose:**

Despite advances in chronic lymphocytic leukemia treatment, effective therapies for cases with Richter’s transformation (RT) are urgently needed. This study aimed to investigate the use of chimeric antigen receptor (CAR)-T cell infusion for RT.

**Methods:**

This study included RT patients at Beijing Boren Hospital between February 2020 and February 2023. Complete response (CR), partial response (PR), overall response rate (ORR), overall survival (OS), progression free survival (PFS), and adverse events were analyzed.

**Results:**

Data from 16 patients with RT who underwent CAR-T cell therapy [62.5% (10/16) males and a median age of 59 years (range: 42–69 years)] were collected. The 3-month CR rate was 56.3% (9/16), with an ORR of 68.8% (11/16). Median follow-up was 19.1 months (4.37-42.48m), the 1-year OS/PFS were 75.0%/68.8%, and the 2-year OS/PFS were 67.5%/61.1%. Among 11 patients with CR (n=9) and PR (n=2), 1 CR case died of an acute coronary event, 10 patients had no recurrence or progression at a median follow-up of 24.6 months. Five patients who did not respond to CAR-T cell therapy had a median OS of only 6.44 months. Univariate logistic regression analysis showed ECOG score [odds ratio (OR)=0.025, P=0.016] were independently associated with ORR. ECOG (OR=40, P=0.016) were independently associated with OS. The presence of an extramedullary mass larger than 5cm did not show statistical significance for overall survival (OS), although the P value was close to 0.05 (OR=15, P=0.051).

**Conclusions:**

CAR-T cell therapy showed potential as a treatment for RT. ECOG score may be independently associated with ORR and OS. RT patients may experience prolonged remission and achieve long-term disease control if they attain remission through chimeric antigen receptor T-cell therapy.

## 1 Introduction

Chronic lymphocytic leukemia (CLL) and small lymphocytic lymphoma (SLL) describe adult hematological disorders that are often considered to be the same disease distinguished by their location (1). This disease is common, it is the most frequent adult leukemia in Western populations, though it is less common in China (2, 3). CLL/SLL has a wide spectrum of progression, in many cases although currently uncurable, progression is slow and the disease is manageable; however, in some cases it progresses rapidly to an aggressive disease (4). Richter’s transformation (RT) is currently defined by the World Health Organization (WHO) as the transition from CLL or SLL to aggressive lymphoma (5). Diffuse large B-cell lymphoma (DLBCL) RT and Hodgkin’s lymphoma (HL) RT are the two major pathologic variants of RT, of which DLBCL-RT is the most prevalent subtype, accounting for about 90% of cases (5, 6).

The therapeutic efficacy of Bruton tyrosine kinase inhibitors (BTKis) and BCL2 inhibitors has been dramatically demonstrated in CLL/SLL patients, significantly boosting both overall survival (OS) and progression-free survival (PFS) (4). However, for those stricken with RT, the prognosis remains distressingly bleak, with median OS spanning merely 9–12 months post-initial diagnosis (7, 8). RT is estimated to occur in around 2-15% of cases of CLL (9). Evidence suggests that specific biomarkers such as those in the DNA damage response pathway can combine with the microenvironment to increase the risk of clonal transformation that leads to RS (10, 11).

Current treatment for RT usually involves immunochemotherapy followed by stem cell transplantation (5). An approach that has shown some benefit in treating patients with refractory B-cell acute lymphoblastic leukemia and non-Hodgkin lymphoma is lymphodepletion chemotherapy followed by chimeric antigen receptor (CAR) modified T cell infusion (12, 13). This involves T-cells that have been genetically modified to express a receptor that recognizes a pre-specified target such as CD19 (14). CAR-T cell therapy has also been reported for RT, but the RT cases form a small part of the study population, or the sample sizes of these studies are small, with a maximum of only 9 RT patients enrolled in one study. The results also varied greatly, probably because of the small sample size (15–17). This was up until the start of 2024, when an international multi-center retrospective study analyzed the clinical data of 69 RT patients who received CAR-T cell therapy at 12 centers. However, this only obtained the results of a median PFS of 4.7 months and a median OS of 8.5 months. The 1-year and 2-year PFS rates were 35.7% and 28.9% respectively, and the 1-year and 2-year OS rates were 42.9% and 38.3% respectively (18). The study data originated from different clinical trials, and the number of cases in each individual center was extremely limited (18). Therefore, more studies are needed.

This retrospective study analyzed RT patients who participated in the CART clinical trial at our center between February 2020 and February 2023, as well as RT patients who received compassionate-use CART cell therapy. Short-term efficacy was assessed, and survival follow-up was performed. The results confirmed that the application of CART cells in RT patients is both safe and effective, and an analysis of influencing factors was conducted.

## 2 Methods

### 2.1 Study design and patient selection

A retrospective study was conducted on patients with RT who participated in any CAR-T cell clinical trial or did not qualify for any clinical trial but underwent homogenous CAR-T cell therapy between February 2020 and February 2023. Eligibility criteria for clinical trial enrollment:(1) relapsed and refractory aggressive B-cell lymphoma with positive target markers (lymphoma cells expressed therapeutic target antigens, such as CD19, CD22, CD20) was diagnosed (standard reference to the National Comprehensive Cancer Network (NCCN) guidelines, 5th edition, 2017 [18]). Relapsed aggressive B-cell lymphoma refers to patients with aggressive B-cell lymphoma who achieved complete response (CR) but have increased tumor burden. Refractory aggressive B-cell lymphoma refers to patients with aggressive B-cell lymphoma who have not achieved CR after 4 courses of standard chemotherapy; (2) patients with assessable disease, including minimal residual disease; (3) aged 18–75 years; (4) predicted survival time more than 3 months; (5) eastern Cooperative Oncology Group (ECOG) performance status score 0–2 points; (6) women of childbearing age who had a negative blood pregnancy test before the start of the study and agreed to take effective contraceptive measures during the study until the last follow-up. The exclusion criteria were: (1) patients diagnosed with primary central nervous system invasive B-cell lymphoma or invasive B-cell lymphoma involving the central nervous system; (2) patients with a history of epilepsy or other central nervous system diseases; (3) patients with active hepatitis B or hepatitis C virus infection, human immunodeficiency virus infection, cytomegalovirus infection, Epstein-Barr virus infection or other uncured active infection; (4) lactating women; (5) people who have used any gene therapy products before. Each participant provided their informed consent. This study was approved by the hospital’s ethics committee (Approval NO: 20191225-PJ-003). During this period, among the patients who were not eligible for clinical trials due to an ECOG score of 3 or central nervous system involvement, if they had a strong willingness for immunotherapy and received CART cell therapy after signing the informed consent form, they were also included in this retrospective study.

### 2.2 CAR-T cell manufacturing and infusion

Patients with negative peripheral blood (PB) tumor cells and less than 10% PB tumor cells had their white blood cells collected directly through leukapheresis. Autologous CD3+ T cells were immunomagnetically selected and then modified with a lentivirus encoding a chimeric antigen receptor.

A chemotherapy regimen without fludarabine/bendamustine was first used to reduce peripheral blood tumor load before lymphocyte collection if the patient’s PB tumor cells were higher than 10%.

Patients with extramedullary tumor loads of 5cm or larger were treated with tumor reduction chemotherapy between lymphocyte collection and CAR-T cell transfusion.

For patients with abnormal lymphocytes found in cerebrospinal fluid (CSF), lumbar puncture was performed and intrathecal injection of methotrexate + cytarabine + dexamethasone was performed until the minimal residual disease (MRD) analysis turned negative by CSF flow cytometry or abnormal lymphocytes were found in the third lumbar puncture. Regardless of whether CSF MRD turned negative, patients with previous CSF abnormalities were given prophylactic intravenous mannitol 125ml twice daily and orally levetiracetam 0.25g twice daily at the time of infusion of CART cells.

A pretreatment scheme based on bendamustine or fludarabine and cyclophosphamide (FC) scheme was adopted before CAR-T cells were infused.

### 2.3 Data collection

Patient age, gender, ECOG scores, prior treatment lines, previous BTKi treatment, TP53 abnormality from fluorescence *in situ* hybridization (FISH)/next generation sequencing (NGS), DLBCL-RT, immunoglobulin heavy chain variable (IGHV) rearrangement negative, extramedullary masses, CSF involvement, BM involvement, PB tumor cell+ were collected retrospectively from the electronic patient records.

The short-term efficacy was evaluated 3 months after transfusion. Extramedullary lesions were evaluated by imaging with a diagnostic-quality computed tomography (CT) scan or positron emission tomography/CT (PET/CT). Bone marrow (BM) puncture morphology and flow cytometry were used to determine remission. If necessary, BM biopsy was performed. Efficacy was assessed according to the Lugano 2014 criteria (Complete response [CR] including complete response in the myelosuppressed state).

Survival follow-up was collected, including CR, partial response (PR), and overall response rate (ORR) defined as CR and PR after CAR-T cell infusion, PFS, and OS. At the same time, the occurrence of cytokine release syndrome, adverse reactions, and outcomes after reinfusion were observed to evaluate safety. The measurement of OS began from the start of CAR-T therapy until the patient’s death or last follow-up. PFS was measured from the initiation of CAR-T treatment to the date of disease progression or death, whichever came first.

### 2.4 Statistical analysis

This study conducted statistical analysis using Stata/SE version 17.0 (Stata Corp., LAA, USA) and Python version 3.9.12 (Visual Studio Code software, Microsoft Corp., WA, USA). Descriptive statistical methods were used to analyze the basic characteristics of the patients. A univariate logistic regression model was employed to explore the association between treatment response and patient characteristics. The Kaplan-Meier method was used to estimate the survival rates and hazard functions for the overall data, and a two-sided log-rank test was applied to compare survival rates and progression-free survival rates between different groups.

## 3 Results

### 3.1 Baseline characteristics

A total of 16 patients with RT who received CAR-T cell therapy were included, all of whom had developed DLBCL (**Table 1**). Among the 16 patients, 11 were from other clinical studies in our center, and the other 5 did not meet the inclusion criteria of previous clinical trials. 3 of The 5 patients had ECOG scores of 3, and tumor cells were detected in lumbar spinal fluid in 3 of them,(1of them ECOG scores was3 and central nervous system involved at the same time), and compassionate CAR-T cell therapy was performed within the time frame of this study. All the five patients who received the same temperament treatment were in that row, and the male had a worse baseline (**Table 1**).

**Table 1:** Patient baseline characteristics.

Total	N=16 (%)	Males:	Females:
Sex		10 (62.5)	6 (37.5)
ECOG	0-3	0-3	0-1
0-1	11 (68.8)	5 (50)	6 (100)
2-3	5 (31.3)	5 (50)	0 (0)
Median age (Years)	59 (42-69)	61 (45-67)	46 (42-69)
DLBCL-RT	16 (100)	10 (100)	6 (100)
Prior treatment lines	5 (2-7)	5 (2-7)	4.5 (3-6)
BTKi failed	15 (93.8)	10 (100)	5 (83.3)
BCL2 inhibitor failed	12 (75.0)	10 (100)	2 (33.3)
Chemotherapy failed	15 (93.8) median 6 cycles	10 (100) median 4 cycles	5 (83.3) median 9 cycles
Universal CAR-T failed	1 (6.25)	1 (10)	0 (0)
Prior auto-transplantation	1 (6.25)	1 (10)	0 (0)
TP53 abnormal at least one of FISH/NGS	9/10 (90)	7/7 (100)	2/3 (67.7)
NGS gene mutations	9/12 (75.0)	7/8 (87.5)	2/4 (50)
FISH deletions/rearrangements	9/13 (69.2)	7/8 (87.5)	2/5 (40)
IGHV rearrangement negative	7/10 (70)	4/6 (66.7)	3/4 (75)
Extramedullary masses	14 (87.5)	10 (100)	4 (66.7)
median maximum diameter (mm)	42.5 (18-144)	44.5 (18-144)	40 (34-50)
≥5cm	4 (25)	3 (30)	1 (16.7)
central masses (MRI)	0 (0)	0 (0)	0 (0)
CSF involvement	3 (18.75)	3 (30)	0 (0)
BM involvement	10 (62.5)	7 (70)	3 (50)
PB tumor cell+	7 (43.8)	5 (50)	2 (33.3)

ECOG, Eastern Cooperative Oncology Group; DLBCL-RT, diffuse large B-cell lymphoma-Richter’s transformation; BTKi, Bruton kinase inhibitor; CAR-T, chimeric antigen receptor T-cell infusion; FISH, fluorescence *in situ* hybridization; NGS, next generation sequencing; IGHV, immunoglobulin heavy chain variable; MRI, magnetic resonance imaging; CSF, cerebrospinal fluid; BM, bone marrow; PB, peripheral blood.

The patients had a median age of 59 years (range: 42–69 years), consisting of 62.5% (10/16) males and 37.5% (6/16) females. The ECOG scores of the patients varied between 0 and 3. The median number of prior treatment lines was 5 (2-7) and 93.8% failed previous BTKi treatment and 75% failed BCL2 inhibitor treatment; 93.8% had undergone chemotherapy (median 6 cycles). One patient had previously failed universal CAR-T cell therapy, and another relapsed after autologous transplantation. NGS detected TP53 gene mutations in 75% (9/12) of cases, and FISH revealed TP53 deletions/rearrangements in 69.2% (9/13) of the cases. Overall, 90% had at least one TP53 abnormality detected by either FISH or NGS. IGHV rearrangement was negative in 70% (7/10) of cases. Extramedullary masses were found in 87.5% (14/16) of patients, with a median maximum diameter of 42.5mm (18-144mm). Masses larger than 5cm were found in 25% (4/16) of cases, marrow involvement was noted in 62.5% (10/16), and the CSF was affected in 18.75% (3/16). For the patients with abnormal lymphocytes found in the CSF, 2–3 rounds of intrathecal injection of methotrexate + cytarabine + dexamethasone pretreatment resulted in negative MRD in the CSF of 2/3 patients before CART infusion, but 1 patient was still positive at infusion. None of the patients had central masses observed in their magnetic resonance imaging (MRI). During lymphocyte collection, 43.8% of patients showed positivity for peripheral blood tumor cells (**Table 1**).

### 3.2 CAR-T cell infusion

For the CAR-T cell infusion approach, 15 of the patients’ CAR-T targets were CD19 (93.75%), one was humanized CD20 (6.25%).

For patients with culture dose below 2×10^6^/kg, the full culture dose was transfused. For patients with a culture dose of 2×10^6^/kg or more, the return dose was selected according to the patient’s condition, and the maximum dose did not exceed 3×10^6^/kg. The actual median infusion dose was 1.37×10^6^/kg (ranging from 0.0059 to 3×10^6^/kg). Three individual culture doses were less than 0.5×10^6^/kg, of which two were 0.1×10^6^/kg and 0.23×10^6^/kg, respectively, and one individual culture was only 3.07×10^3^/kg. The patient with a culture dose of only 3.07×10^3^/kg was re-cultured once, but still only 2.83×10^3^/kg of CAR-T cells were obtained, so the total return dose for this patient was 0.0059×10^6^/kg.

### 3.3 CAR-T cell expansion

Only 1 patient, whose infusion dose was less than 0.1×10^6^/kg, did not achieve detectable CAR-T cell expansion *in vivo* after transfusion, the other 15 patients had CAR-T cell expansion in peripheral blood after transfusion. The median peak amplification time appeared on D11 (D7-D15) after reinfusion, and the median peak amplification in CAR-T cells was 60.1×10^6^/L (0.144×10^6^/L-7150 ×10^6^/L). Due to the fact that most patients lived too far from the hospital and other reasons, the monitoring of the number of peripheral blood CAR-T cells was stopped during the period when the CAR-T examination might still provide useful information, because they were transferred to a local hospital for re-examination. The re-examinations in the local hospitals included the survival and remission status, but the CAR-T survival time of all patients could not be counted. Peripheral blood CAR-T cells were monitored regularly in only 3 patients. In one of them the peripheral blood CAR-T cells were most positive on D181 and were negative after reexamination, but peripheral CAR-T cells could still be detected in the other two patients at their last re-examination, on D378 and D1116, respectively, and their next follow-up appointment has not yet arrived.

**Figure 1:**
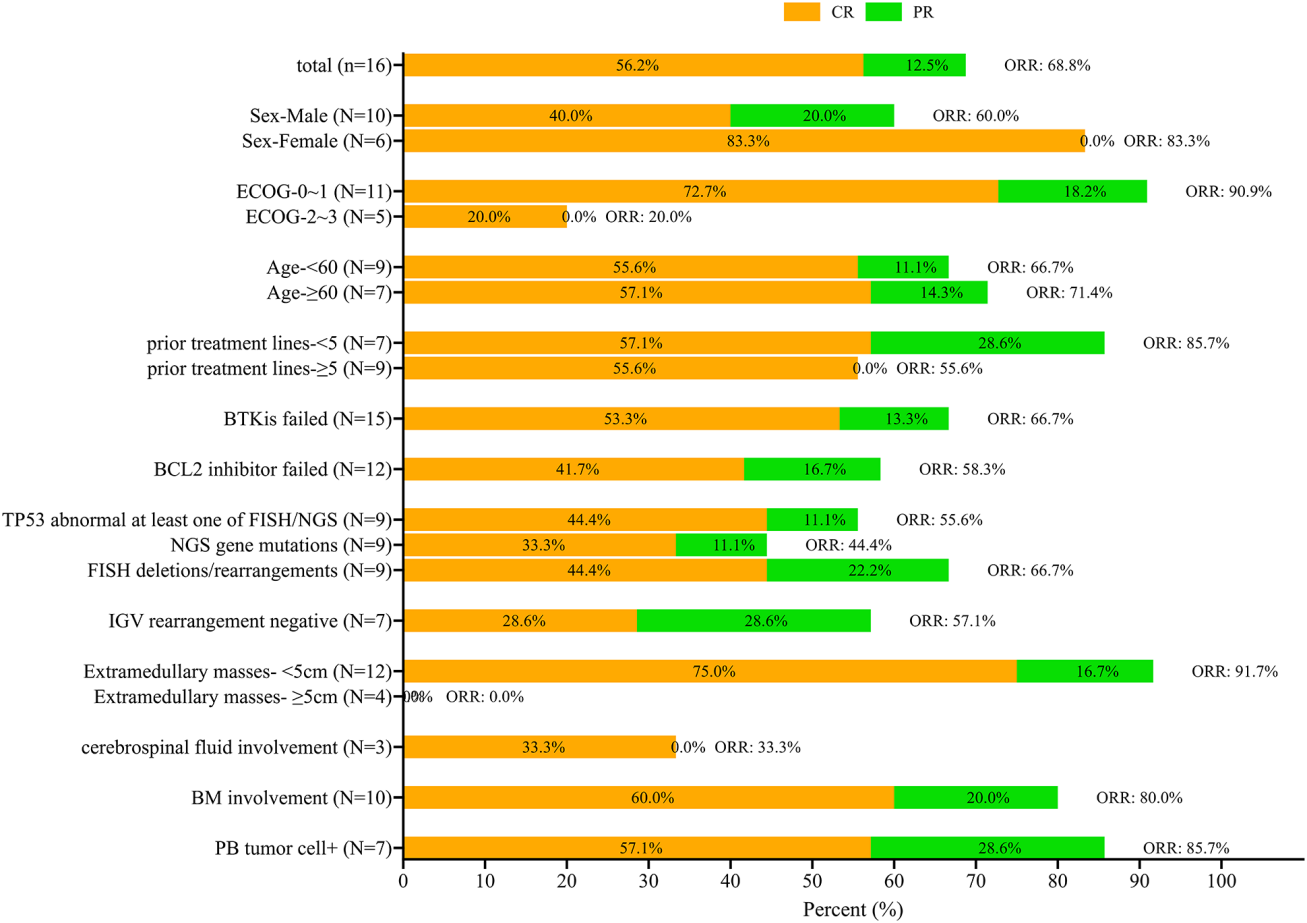
Survival rates at 3-month follow-up according to different baselines. The complete response (CR) is indicated in orange and partial response (PR) in green with the total overall response rate (ORR) also indicated.

Short-term therapeutic effect of 3 months (**Figure 1**).

The 3-month CR and ORR according to different baselines are shown in **Figure 1**. The CR rate at the 3-month mark stood at 56.3% (9/16), with an ORR of 68.8% (11/16). The CR and ORR of male patients were 40% and 60%, respectively, and that of female patients were 83.3% and 83.3%, respectively. In patients with an ECOG score of 0 to 1, CR and ORR were 72.7% and 90.9%, while in patients with an ECOG score of 2-3, CR and ORR were only 20%. Patients with dual resistance to BCL2 inhibitor and BTKi had lower CR and ORR than those with simple BTKi resistance (CR 41.7% and ORR 58.7% vs CR 53.3% and ORR 66.7%). Patients with negative IGHV rearrangement had lower CR and ORR, with a CR rate of only 28.6% and a lower ORR of 57.7%. None of the four patients with extramedullary masses larger than 5cm achieved ORR. Only 1 of 3 patients with central involvement achieved CR (33.3%), while the other 2 did not respond to CAR-T therapy (**Figure 1**).

### 3.4 Long-term efficacy

**Figure 2:**
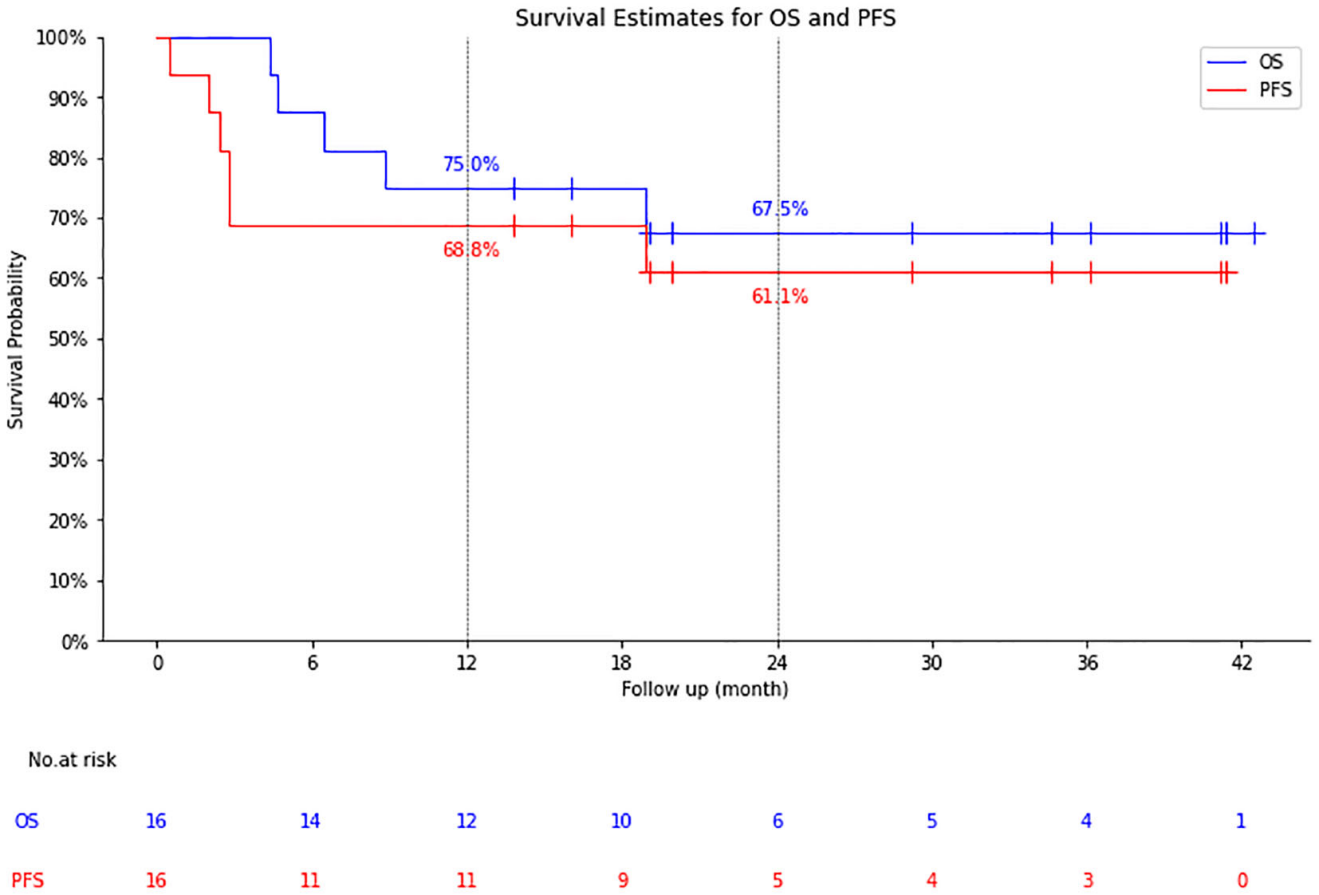
Survival estimates for overall survival (OS) and progression free survival (PFS).

The median follow-up time was 19.1 months (4.37-42.48 months), the 1-year OS/PFS were 75.0% and 68.8%, and the 2-year OS/PFS were 67.5% and 61.1%, respectively. Median OS and PFS were not reached (**Figure 2**).

**Figure 3:**
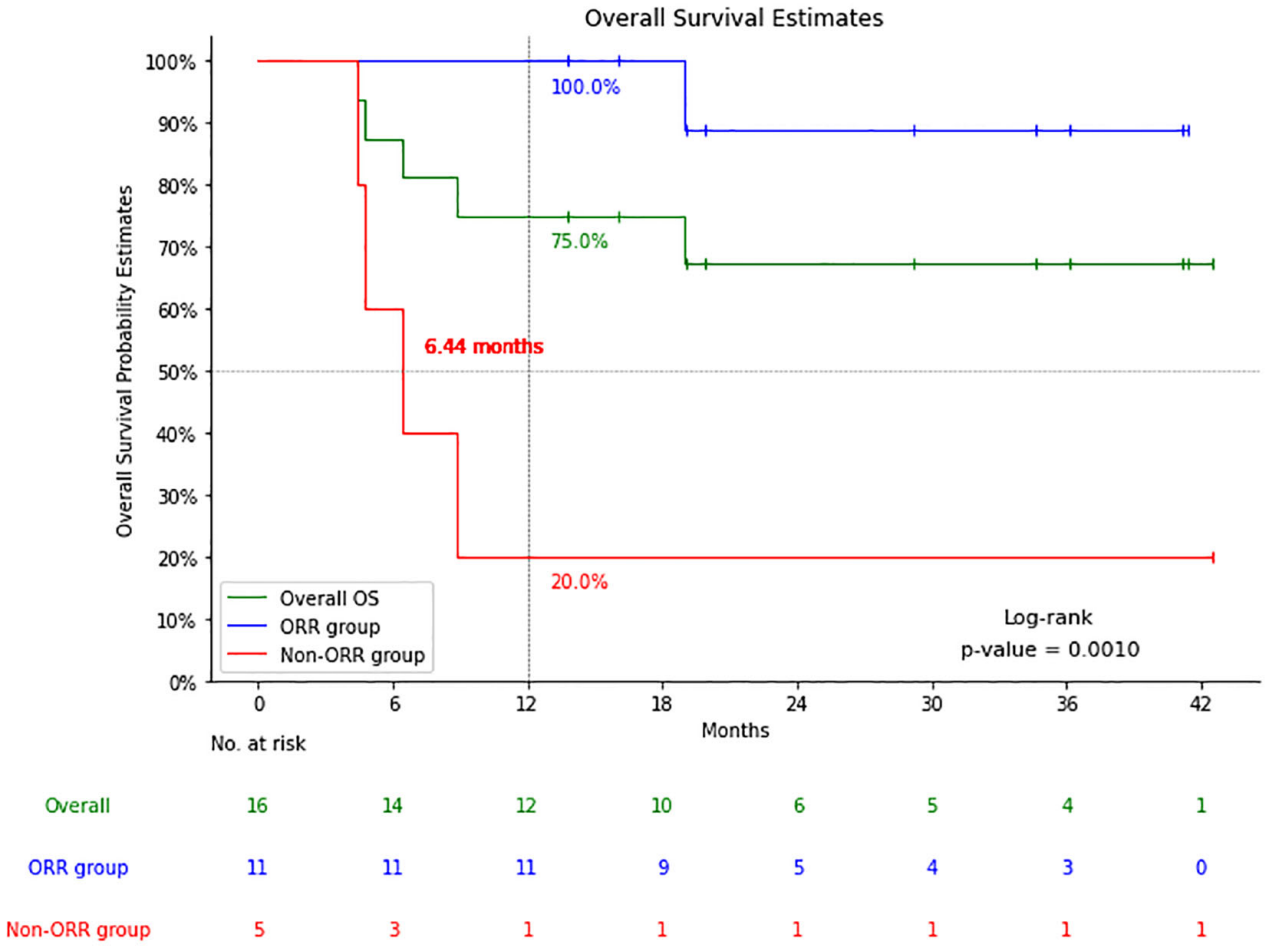
Comparison of overall survival (OS) of patients with a response (cases with complete response (CR) and partial response (PR): the Response group) and patients with no response (cases with stable disease (SD) or progressive disease (PD): the Non-response group).

Among 11 patients with CR (n=9) and PR (n=2), 1 with CR died of an acute coronary event 19 months after CAR-T cell infusion, the remaining 10 patients had no recurrence or progression at a median follow-up of 24.6 months (**Figure 3**).

The five patients who did not respond to CAR-T cell therapy had a median OS of only 6.44 months. One of the patients who did not respond to CAR-T cell therapy was treated with the other two target CAR-T cell therapies separately but was still unable to achieve complete remission until allogeneic transplantation combined with the same donor CAR-T cell therapy later achieved complete remission, the patient has been followed up for 38 months, and is still alive today. The other four, despite attempts at salvage treatment including targeted drugs combined with chemotherapy and second CAR-T cell therapy, all died, with a median OS of only 5.6 months (4.4-8.9m).

### 3.5 Analysis of influencing factors

A univariate logistic regression model was used to analyze the effects of age, gender, ECOG, CSF/BM involvement, 5cm or greater mass, and CAR-T cell infusion dose on PFS/OS and CR/ORR 3 months after infusion. Only ECOG score had a significant effect on ORR (odds ratio (OR)=0.025, p=0.016), and the risk factors affecting the timing of OS were ECOG (OR=40, P=0.016). The presence of an extramedullary mass larger than 5cm did not show statistical significance for overall survival (OS), although the P value was close to 0.05 (OR=15, P=0.051). No factors had a significant effect on CR rate and PFS (**Table 2**).

**Table 2:** Univariate logistic regression model.

baseline characteristics	CR (3months)		ORR (3months)		PFS		OS	
	Odds ratio	P	Odds ratio	P	Odds ratio	p	Odds ratio	p
Age (<60/≥60)y	0.997	0.978	1.029	0.622	0.997	0.955	1.037	0.544
Sex (male/female)	0.133	0.113	0.300	0.344	0.200	0.203	0.300	0.344
ECOG (2-3/0-1)	-2.367	0.070	0.025	0.016	------	------	40.00	0.016
CSF involvement (+/-)	0.313	0.978	0.150	0.172	------	------	6.667	0.172
BM involvement (+/-)	1.500	0.697	4.000	0.223	0.429	0.428	0.857	0.889
block size≥5cm (+/-)	------	------	------	------	------	------	15.00	0.051
CART infusion dose*	3.144	0.978	4.000	0.223	0.513	0.271	0.782	0.672
PB tumor cell** (+/-)	2.625	0.383	6.750	0.112	0.250	0.223	0.5625	0.613

* CART infusion dose every increase 1 x 10 ^ 6 / kg; **Whether tumor cells were still present in peripheral blood at the time of lymphocyte collection.

## 4 Safety assessment

### 4.1 Cytokine release syndrome

occurred in 75.0% (12/16) of patients, of which, 62.5% (10/16) were grade 1–2 and 12.5% (2/16) were grade 3. Central CRS occurred in 6.3% (1/16) of patients (**Table 3**). The median onset time and resolution time of the CRS reaction were day 0 and day 10, respectively, with a median duration of 8.5 days. Of the patients with CRS reaction, 6 were treated with hormone therapy, and 3 of them were treated with interleukin (IL)-6 monoclonal antibody or IL-6 receptor monoclonal antibody. The remaining 6 cases were treated only with non-steroidal antipyretic analgesics.

**Table 3:** Safety analysis.

Patients with cytokine release syndrome (CRS)	N (%)	Male (%)	Famale (%)
Total	12 (75.0)	7 (70)	5 (83.3)
Grade 1	9 (56.3)	5 (50)	4 (66.7)
Grade 2	1 (6.3)	0 (0)	1 (16.7)
Grade 3	2 (12.5)	2 (20)	0 (0)
Grade 4	0 (0)	0 (0)	0 (0)
Time to cytokine release syndrome onset, days	0 (0-4)	0 (0-4)	1 (0-1)
Time to cytokine release syndrome resolution, days	10 (6-41)	11 (6-41)	10 (7-17)
Duration of CRS (day,range)	8.5 (6-41)	8 (6-41)	10 (6-16)
Patients with neurological events	1 (6.3)	1 (10)	0 (0)
Time to CRS onset, days	3	3	
Time to CRS resolution, days	9	9	
Medication for cytokine release syndrome			
Corticosteroids	6 (37.5)	4 (40)	2 (33.3)
Monoclonal antibody	3 (18.8)	2 (20)	1 (16.6)
Infections			
Pulmonary infections	3 (18.8)	3 (30)	0 (0)
Intestinal infection	5 (31.3)	2 (20)	3 (50)
COVID-19 infection	1 (6.3)	1 (10)	0 (0)
Perianal infection	1 (6.3)	1 (10)	0 (0)
Other adverse events of special interest			
Agranulocytosis>15days	7 (43.8)	4 (40)	3 (50)
Tumor lysis syndrome	0 (0)	0 (0)	0 (0)
Second primary malignancy	0 (0)	0 (0)	0 (0)
Hypogammaglobulinemia	10 (62.5)	6 (60)	4 (66.7)

### 4.2 ICANS

Only 1 patient (6.3%) developed a central CRS response on day 3 of CAR-T cell infusion(**Table 3**). This presented with uncontrolled waving of the upper limbs, slanted upward gaze of the eyes, abnormal contraction of facial muscles, cognitive and speech impairment, and no affected vital signs. After mannitol and hormone therapy, the upper limb flapping and abnormal facial muscle contraction lasted only a few minutes, while the cognitive and speech impairment lasted for 6 days before gradual and complete recovery. Prior to CAR-T cell infusion in this patient, no central mass was observed on enhanced MRI and no tumor cells were detected on CSF examination.

### 4.3 Other adverse reactions

During treatment, 5 patients developed an intestinal infection, 3 a pulmonary infection, 1 COVID-19 infection, and 1 perianal infection. Agranulocytosis was found in 7 patients (43.8%) more than 15 days after treatment, and gammaglobulinemia (62.5%) in 10 patients. No tumor lysis syndrome was found in any of the patients, and no second tumor was found during follow-up (**Table 3**).

## 5 Discussion

### 5.1 The efficacy of CART for RT patients has been proved

The results showed that the 16 patients with RT who received CAR-T cell therapy had 56.3% 3-month CR rate with an ORR of 68.8%. The 1-year PFS was 68.8% and the 2-year PFS was 61.1%. CR was achieved in 9 cases and PR in 2 cases, 1 case with CR case died of an acute coronary event, but 10 patients had no recurrence or progression at a median follow-up of 24.6 months. The 5 patients who did not respond to CAR-T cell therapy had a median OS of 6.44 months. The results of this study suggest that CAR-T cell therapy shows potential for patients with RT. Of the 16 patients in this study, followed up for a median of 19.1 months after CAR-T cell therapy, the median OS was still not reached, which was significantly higher than the median survival of 9–12 months since diagnosis of RT reported in previous studies (7, 8).

Most studies investigating CAR-T cell therapy for RT are limited by their small sample size. One of the larger studies included 24 patients with CLL achieved an ORR of 71% which compares well with our ORR of 68.8% (16). However, only five of the patients had RT in that study, and in those patients 2 cases had CR, 1 had PR, while 2 patients had progressive disease (16). Another study included 9 patients with RF achieved an ORR of 100% in the 8 patients that could be fully evaluated (17). A study of 8 patients with B cell malignancies treated with CAR-T cell infusion included 2 patients with RS, of these two cases 1 achieved PR with progression within 2 months of treatment (19). The results of these studies varied widely, and the follow-up time was very limited. The largest international multicenter retrospective study on data was an analysis of 69 RT patients in 12 households, featuring an ORR of 63.8% and a CR of 46%, similar to our response rates. However, after a median follow-up of 24 months in that study, the median PFS and OS were merely 4.6/8.5 months, while after a median follow-up of 19.1 months in our study, the median PFS and OS were not reached (19). The results of our study add information on an additional 16 patients with RT and suggest that CAR-T has potential as RT therapy. According to the survival follow-up of 11 patients who responded to CAR-T cell therapy, except for 1 patient who suffered sudden cardiac death at 19 months after reinfusion, the remaining 10 patients did not have relapse or disease progression during a median follow-up of 24.6 months. Previous studies have also discovered that RT patients who achieved CR after CAR-T cell therapy had an extremely low recurrence rate over 18 months (only one case had a late relapse at 23 months) (19). After extended follow-up time in the future, it is likely that a longer CR status can be maintained, and the possibility of cure cannot be excluded.

### 5.2 CART cell therapy can still improve the prognosis of RT even if the TP53 gene is abnormal

In this study, most patients had abnormalities in the TP53 gene as identified by NGS or FISH. TP53 mutations predict resistance to chemoimmunotherapy and a shorter time to progression in CLL (20). TP53 is also a driver of RT (21). Unfortunately, limitations with the study sample size meant that the effect of TP53 mutation on CAR-T cell therapy outcomes could not be directly investigated in this study. However, such a high level of TP53 abnormalities in this series with a reasonable effectiveness of CAR-T cell therapy indirectly indicates that cases with RT with abnormal TP53 can benefit from this approach.

### 5.3 Adverse reactions such as CRS are controllable

Another important aspect of RT treatment is the occurrence of adverse events (22). CRS is a common event during CAR-T cell therapy that can develop into a serious complication (23, 24). In this series, although the incidence of CRS response reached 75%, there was no grade 4–5 CRS response, and the safety was acceptable. For comparison, in one of the previous studies of CAR-T cell therapy in CLL, CRS was seen in 83% (18 with grade ½, one with grade 4 and one with grade 5) (16). In the previously mentioned multicenter retrospective study of 69 patients with RT treated with CAR-T cells, 4 cases of CAR-T-related death occurred (19).

In this study, central CRS reaction occurred in only 1 patient, but no abnormal lymphocytes were found in the CSF before treatment in this patient, while no central CRS reaction was found in 3 patients with tumor cells found in the CSF, which was considered to be related to the intrathecal triple drug injection and preventive administration of mannitol and levetiracetam in patients with abnormal CSF before treatment. Therefore, the safety of CAR-T cell therapy for RT is controllable. Central CRS reaction can be prevented by lumbar puncture, intrathecal injection and reinfusion with mannitol and levetiracetam.

Bridging pretreatment for those with a large tumor burden and central ICANS prevention for patients with central involvement may improve our safety. Compared with the multi-center report of 69 cases, the improvement in safety during our CART treatment period led to not losing too many patients in the early stage, thus preventing our PFS/OS from being achieved (19).

### 5.4 Look forward to the future

CAR-T cell therapy is continuing to develop (25). The generation of CARs involves artificial receptors produced to specifically target antigens expressed on the cell surface (26). In this study most patients received CAR-T cell therapy that targeted CD19, which has been a promising target in CLL (14). However, it has been suggested that novel targets are still needed as some patients develop resistance to CD19 CAR-T cells from antigen loss. One patient in this series had CD20 targeted CAR-T cell therapy which has begun to be developed as a target for mature B cell malignancies, including CLL (14). Other approaches such as biomarker assessment to predict patient outcomes might also be of benefit (27). It seems likely that as the CAR-T cell therapy approach develops in CLL outcomes will improve (28, 29), including for patients with RT (30).

### 5.5 Limitations

This study has some limitations. As described above, while this study is one of the largest involving RT patients at single center, the case numbers were still limited. The limited number of patients did not allow for multivariate analysis. Also due to the number of cases, univariate analysis only found that ECOG had statistical significance for ORR and OS, and no other statistically significant factors were found. The presence of an extramedullary mass larger than 5cm did not show statistical significance for overall survival (OS), although the P value was close to 0.05. Although it can be seen from **Figure 1**, none of the patients with extramedullary masses larger than 5cm achieved CR or PR. However, univariate analysis showed that it had no statistical difference in 3-month ORR/CR rate and PFS. The patients included in this study were not limited to those with an ECOG score of 0-1, but also included 5 patients (31.3%) with an ECOG 2–3 score. The difference in survival was mainly considered to be treatment-related, but further studies in larger populations from multiple treatment centers will provide more urgently required information. The study was also limited by the medium-term follow-up time. Long-term follow-up will provide more information on the survival rates and true effectiveness of CAR-T cell therapy to provide a cure for RT.

Univariate logistic regression analysis suggested that ECOG was related to ORR, while ECOG was potential risk factors associated with OS. No factors had significant effects on CR rate or PFS. Therefore, this study suggests that CAR-T cell therapy shows potential as a therapy for RT, but outcomes are influenced by ECOG score. Extramedullary mass with a diameter of >5cm did not show statistical significance for OS, but it may also become an important influencing factor after expanding the sample size.

The therapeutic effect of female patients was superior to that of male patients from the perspective of swimlane diagrams, and no statistical difference was found in univariate analysis. However, all the 5 patients with homotropic treatment who did not meet the requirements of the clinical trial were male, which led to an imbalance in the baseline situation between men and women and might cause differences in therapeutic effects between men and women. Due to the small amount of data, it cannot fully indicate that the general condition of male patients is worse after Richter transformation.

In conclusion, The aim of this study was to present a case series of patients with RT who received CAR-T cell therapy to provide more evidence of the effectiveness of CAR-T cell therapy for RT. CAR-T cell therapy showed potential as a treatment for RT. ECOG score may be independently associated with ORR and OS. A multi-center large sample randomized trial is needed to verify the results of this study. Analysis of ORR and PFS in this case series of 16 patients suggests that CAR-T cell therapy was effective in patients with RT, even though most of them are accompanied by TP53 gene or FISH abnormality, especially in patients with ECOG0–1 score, which can be expected to cure some patients. However, RT patients who do not respond to CAR-T cell therapy have poor prognosis and short survival. The same conclusion as in previous literature is that RT patients may experience prolonged remission and achieve long-term disease control if they attain remission through chimeric antigen receptor T-cell therapy. The median follow-up was 19.1 months, and the median PFS and OS were not reached, which may be related to the reduction of tumor cell burden in peripheral blood before lymphocyte collection and bridge therapy before transfusion.

## Data Availability

The original contributions presented in the study are included in the article/supplementary material. Further inquiries can be directed to the corresponding author/s.
